# CD161^+^ CD4^+^ T Cells Harbor Clonally Expanded Replication-Competent HIV-1 in Antiretroviral Therapy-Suppressed Individuals

**DOI:** 10.1128/mBio.02121-19

**Published:** 2019-10-08

**Authors:** Xiaomin Li, Zhaoli Liu, Qijuan Li, Ronglin Hu, Lu Zhao, Yanyan Yang, Jiacong Zhao, Zhuoqiong Huang, Hongbo Gao, Linghua Li, Weiping Cai, Kai Deng

**Affiliations:** a Institute of Human Virology, Key Laboratory of Tropical Disease Control of Ministry of Education, Zhongshan School of Medicine, Sun Yat-sen University, Guangzhou, China; b Department of General Surgery, Guangzhou First People’s Hospital, The Second Affiliated Hospital, South China University of Technology, Guangzhou, China; c Infectious Disease Center, Guangzhou Eighth People’s Hospital, Guangzhou Medical University, Guangzhou, China; d Program of Pathobiology, Fifth Affiliated Hospital, Zhongshan School of Medicine, Sun Yat-sen University, Guangzhou, China; Ragon Institute of MGH, MIT and Harvard; Johns Hopkins Bloomberg School of Public Health

**Keywords:** HIV-1, latent reservoir, CD4^+^ T cells, CD161, clonal expansion, HIV-1, latent reservoir

## Abstract

The presence of an extremely stable latent reservoir of HIV-1 is the major obstacle to eradication, despite effective antiretroviral therapy (ART). Recent studies have shown that clonal expansion of latently infected cells without viral reactivation is an important phenomenon that maintains the long-term stability of the reservoir, yet its underlying mechanism remains unclear. Here we report that a subset of CD4^+^ T cells, characterized by CD161 expression on the surface, is highly permissive for HIV-1 infection. These cells possess a significantly higher survival and proliferative capacity than their CD161-negative counterparts. More importantly, we found that these cells harbor HIV-1 DNA and replication-competent latent viruses at a significantly higher frequency. By using massive single-genome proviral sequencing from ART-suppressed individuals, we confirm that CD161^+^ CD4^+^ T cells contain remarkably more identical proviral sequences, indicating clonal expansion of the viral genome in these cells. Taking the results together, our study identifies infected CD161^+^ CD4^+^ T cells to be a critical force driving the clonal expansion of the HIV-1 latent reservoir, providing a novel mechanism for the long-term stability of HIV-1 latency.

## Abstract (precis)

The latent reservoir continues to be the major obstacle to curing HIV-1 infection. The clonal expansion of latently infected cells adds another layer maintaining the long-term stability of the reservoir, but its mechanism remains unclear. Here, we report that CD161^+^ CD4^+^ T cells serve as an important compartment of the HIV-1 latent reservoir and contain a significant amount of clonally expanded proviruses. In our study, we describe a feasible strategy that may reduce the size of the latent reservoir to a certain extent by counterbalancing the repopulation and dissemination of latently infected cells.

## INTRODUCTION

Antiretroviral therapy (ART) effectively halts HIV-1 replication but still fails to eradicate the viruses (1, 2). The vast majority of HIV-1-infected individuals experience a rapid rebound of viremia once treatment is interrupted (3). Numerous layers of evidence have shown that the persistence of HIV-1 is due to the presence of a remarkably stable latent reservoir of HIV-1, primarily in resting memory CD4^+^ T cells. A quantitative viral outgrowth assay (QVOA) estimated that the half-life of these latently infected cells is 44 months (4), making it almost impossible to obtain a cure by relying just on ART. Therefore, the latent reservoir is widely considered the major barrier to curing HIV-1 infection (5, 6). The dynamic mechanisms that maintain long-term immune memory within the CD4^+^ T cell compartment may play an important role in the stability and longevity of the HIV-1 latent reservoir (7). Recently, several reports have suggested that the latent reservoir is maintained through cellular proliferation and the clonal expansion of infected CD4^+^ T cells in ART-treated HIV-1-infected individuals (8, 9). Further studies also indicated that infected CD4^+^ T cells harboring replication-competent provirus can undergo homeostatic proliferation in response to T cell receptor (TCR) stimulation or certain cytokines without viral reactivation (10, 11). Mathematical modeling showed that clonal expansion represents a critical factor contributing to the slow decay of the latent reservoir (12). However, the mechanisms underlying the expansion of latent HIV-1 remain largely unknown. Considering that HIV-1 proviruses are not equally distributed across memory CD4^+^ T cell subsets (11), we are curious to know and it is important to know whether a certain CD4^+^ T cell subset acts as a major driving force for the clonal expansion of latently infected CD4^+^ T cells.

Previous reports have suggested that the relatively long-lived Th17 cells may contribute to HIV-1 persistence under ART (13, 14). As Th17 cells are derived from CD161^+^ blood precursors (15, 16), CD161 would be an efficient marker to easily identify Th17 cells (15, 17, 18). Another study proposed that a subset of memory CD8^+^ T cells defined phenotypically by the expression of high levels of CD161 has stem-like properties (19). Interestingly, studies have also identified the existence of CD4^+^ T cells with stem-like properties among Th17 cells, suggesting that cell fate diversification results in the generation of T cells with a stem-like phenotype, even within more differentiated T cell subsets (20, 21). In addition, CD161 can act as a costimulatory receptor to increase the response to TCR stimulation and is a marker for long-lived antigen-specific memory T cells (17, 22–24). Based on the aforementioned characteristics of CD161^+^ CD4^+^ T cells, we hypothesize that these cells may play a critical role in the expansion of the HIV-1 latent reservoir. In this study, we describe the existence of a specialized subset of CD4^+^ T cells defined phenotypically by the expression of CD161 on their surfaces. These cells are more susceptible to HIV-1 infection and have a higher proliferative ability. Additionally, larger amounts of latent HIV-1 are presented within this subset than in CD161^−^ CD4^+^ T cells. More importantly, CD4^+^ T cells harboring clonally expanded HIV-1 proviral sequences are significantly enriched within the CD161^+^ subset from ART-treated infected individuals than within other CD4^+^ T cells, suggesting that HIV-1-infected CD161^+^ CD4^+^ T cells may indeed drive the clonal expansion of latently infected cells.

## RESULTS

### CD161^+^ CD4^+^ T cells are highly permissive for HIV-1 infection.

**FIG 1:**
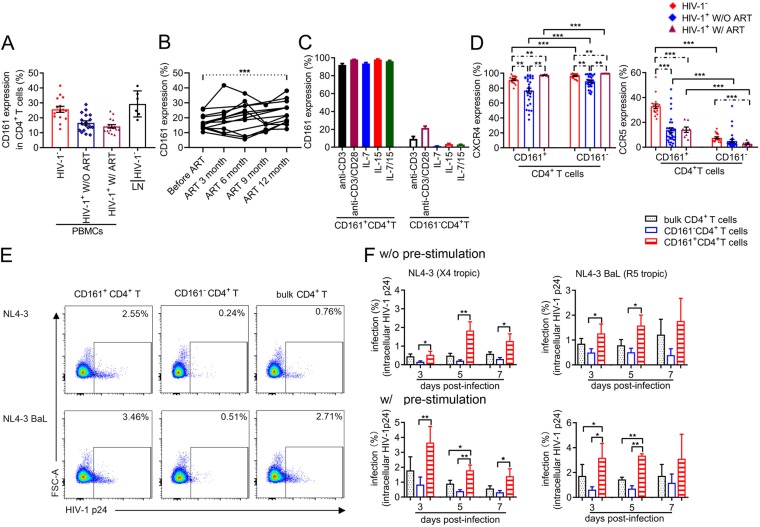
CD161^+^ CD4^+^ T cells are highly permissive for HIV-1 infection. (A) Percentage of CD161-positive subsets among CD4^+^ T cells in blood or lymph node. HIV-1^−^, results for samples from 15 HIV-seronegative donors; HIV-1^+^ W/O ART, results for samples from 18 viremic patients not on ART; HIV-1^+^ W/ ART, results for samples from 16 HIV-1-infected subjects with an undetectable plasma viral load on ART and CD4 cell counts above 450 per μl; LN HIV-1^−^, lymph node tissue samples from 6 HIV-1-negative individuals. (B) Percentage of CD161-positive subsets in CD4^+^ T cells from the beginning of ART to 12 months after the start of treatment (*n* = 13). (C) Expression level of CD161 on sorted CD161-positive or -negative CD4^+^ T cells from healthy donors (*n* = 4) under different treatment conditions for 12 days. (D) Frequency of cells expressing CXCR4 (left) and CCR5 (right) within CD161-positive and negative CD4^+^ T cell subsets. (E and F) Infectivity of X4- or R5-tropic HIV-1 for bulk, CD161^+^, or CD161^−^ CD4^+^ T cells. Subsets from HIV-negative subjects were sorted and then exposed directly (without prestimulation) to R5-tropic NL4-3 (BaL *env*) (*n* = 6) or X4-tropic NL4-3 (*n* = 11) or were first prestimulated via anti-CD3/CD28 for 3 days and then exposed to NL4-3 (BaL *env*) (*n* = 4) or NL4-3 (*n* = 6). Intracellular HIV-1 p24 levels were quantified by FACS (E) at the indicated time points postinfection (F). FSC-A, forward scatter area. Significant intergroup differences were determined using the rank Kruskal-Wallis test incorporating Dunn’s tests for multiple comparisons. The Mann-Whitney U test or Student's *t* test was used to compare the statistical significance between cell subsets. *P* values less than 0.05 were considered significant. *, *P < *0.05; **, *P < *0.01; ***, *P < *0.001.

To investigate whether HIV-1 infection affects CD161^+^ CD4^+^ T cells, we measured the percentage of cells expressing CD161 among CD4^+^ T cells from the blood or lymph node (LN) of HIV-1-negative and HIV-1-positive individuals receiving or not receiving ART. The sociodemographic, clinical, and behavioral characteristics of the individuals studied are presented in Table 1. In healthy individuals, a median of 26% of total CD4^+^ T cells (interquartile range [IQR], 21 to 30%) expressed CD161, which was 1.5 times higher than the proportion in HIV-1-infected individuals not on ART (median, 16%; IQR, 12 to 22%) and 1.8 times higher than that in HIV-1-infected donors on ART (median, 14%; IQR, 11 to 17%) (Fig. 1A), suggesting that HIV-1 infection may deplete or downregulate CD161^+^ CD4^+^ T cells. The depletion or downregulation of CD4^+^ T cells may be associated with high levels of immune activation in HIV-1-infected subjects (25, 26). Our results showed that the activation level (CD25, CD69, CD38, or HLA-DR expression) on CD161^+^ CD4^+^ T cells was higher in HIV-1-infected subjects than in healthy donors (see Fig. S1A in the supplemental material). To further clarify whether the decreased CD161 expression in HIV-1-infected individuals was really caused by HIV-1 infection and to determine the proportion of CD161^+^ CD4^+^ T cells that could be recovered after ART, we followed CD161 expression on CD4^+^ T cells from 13 subjects at different time points from the beginning of ART to after 1 year of ART. Our results showed that the proportion of CD161-positive cells increased significantly after 1 year of ART (Fig. 1B; clinical information is presented in Table 2), suggesting that these cells may indeed be affected by HIV-1 replication. To check whether the expression of CD161 is stable, we applied TCR activation and a T cell homeostasis signal to CD161-positive and -negative cells. Our data showed that CD161 expression on CD4^+^ T cells was relatively stable, as CD3/CD28 activation or the level of the interleukin-7 (IL-7)/IL-15 signal did not change for either subset (Fig. 1C), supporting the suggestion that CD161 may be used as a marker to identify this specific cell population.

**TABLE 1:** Clinical characteristics of the HIV-1-infected donors involved in this study^*a*^

Sample identifier	Age (yr)	Sex	Cell count (no. of cells/μl)		Viral load (no. of copies/ml)	Time of infection before initiation of therapy (mo)	Time on ART (mo)	Therapeutic regimen	Figure(s) or table where the subject was involved
			CD4^+^ T cells	CD8^+^ T cells					
1	23	M	321	822	1.68E+04	0	0	t.n.	Fig. 1A and C; Fig. S1A
2	41	M	437	6,469	1.69E+05	0	0	t.n.	Fig. 1A and C; Fig. S1A
3	30	M	230	944	1.50E+05	0	0	t.n.	Fig. 1A and C; Fig. S1A
4	24	F	250	834	ND	0	0	t.n.	Fig. 1A and C; Fig. S1A
5	41	M	119	856	ND	0	0	t.n.	Fig. 1A and C; Fig. S1A
6	27	M	107	685	ND	0	0	t.n.	Fig. 1A to C; Fig. S1A
7	29	M	304	771	1.58E+04	0	0	t.n.	Fig. 1A to C; Fig. S1A
8	33	M	286	738	5.87E+03	1	0	t.n.	Fig. 1A to C; Fig. S1A
9	27	M	100	616	5.64E+04	1	0	t.n.	Fig. 1A to C; Fig. S1A
10	55	M	267	1,267	ND	0	0	t.n.	Fig. 1A to C; Fig. S1A
11	27	M	280	518	6.49E+03	1	0	t.n.	Fig. 1A to C; Fig. S1A
12	24	M	291	782	ND	0	0	t.n.	Fig. 1A to C; Fig. S1A
13	36	M	166	909	ND	0	0	t.n.	Fig. 1A to C; Fig. S1A
14	43	F	250	724	ND	1	0	t.n.	Fig. 1A to C; Fig. S1A
15	39	M	65	658	ND	1	0	t.n.	Fig. 1A to C; Fig. S1A
16	37	M	102	402	1.55E+05	0	0	t.n.	Fig. 1A to C; Fig. S1A
17	49	M	45	308	ND	0	0	t.n.	Fig. 1A to C; Fig. S1A
18	18	F	249	573	ND	0	0	t.n.	Fig. 1A to C
19	28	M	724	240	<50	1	55	ABC + 3TC + EFV	Fig. 1A and C and 4A
20	50	F	469	1,050	<50	0	26	AZT + 3TC + NVP	Fig. 1A and C and 4A; Fig. S3B
21	44	M	551	520	<50	2	38	AZT + 3TC + EFV	Fig. 1A and C and 4A; Fig. S3B
22	30	M	487	560	<50	2	46	TDF + 3TC + EFV	Fig. 1A and C and 4A; Fig. S3B
23	24	M	484	750	<50	11	29	TDF + 3TC + EFV	Fig. 1A and C and 4A; Fig. S3B
24	38	M	823	670	<50	1	33	TDF + 3TC + EFV	Fig. 1A and C and 4A; Fig. S3B
25	71	M	590	460	<50	0	36	d4T + 3TC + EFV	Fig. 1A and C; Fig. S3A and B
26	60	M	818	760	<50	1	44	TDF + 3TC + EFV	Fig. 1A and C; Fig. S3A and B
27	43	M	491	580	<50	0	53	TDF + 3TC + EFV	Fig. 1A; Fig. S3A and B
28	32	M	727	616	<50	5	49	AZT + 3TC + EFV	Fig. 1A; Fig. S3A and B
29	29	M	622	812	<50	0	38	AZT + 3TC + EFV	Fig. 1A; Fig. S3A and B
30	38	F	574	599	<50	2	27	AZT + 3TC + NVP	Fig. 1A; Fig. S3A and B
31	27	F	918	629	<50	1	25	TDF + 3TC + LPV/r	Fig. 1A; Fig. S3A and B
32	46	F	818	865	<50	0	51	FTC + TDF + LPV/r	Fig. 1A; Fig. S3A and B
33	35	F	616	984	<50	3	44	AZT + 3TC + EFV	Fig. 1A; Fig. S3A and B
34	42	M	976	867	<50	3	109	d4T + 3TC + EFV	Fig. 1A; Fig. S3A and B
35	39	F	564	1,020	<50	31	120	d4T + 3TC + NVP	Fig. 2C
36	48	M	525	847	<50	0	138	d4T + 3TC + NVP	Fig. 2C
37	40	M	501	1,004	<50	20	101	d4T + 3TC + NVP	Fig. 2C
38	43	M	1,002	613	<50	1	79	TDF + 3TC + EFV	Fig. 2C
39	75	M	489	1,062	<50	121	123	d4T + 3TC + NVP	Fig. 2C
40	37	M	583	1,352	<50	0	79	AZT + 3TC + EFV	Fig. 2C
41	43	M	514	719	<50	47	88	TDF + 3TC + EFV	Fig. 2C and 3C and D
42	33	M	787	657	<50	19	63	TDF + 3TC + EFV	Fig. 2C and 3C and D
43	61	F	496	792	<50	47	94	AZT + 3TC + EFV	Fig. 3B and F and 4A
44	40	M	687	803	<50	0	27	TDF + 3TC + EFV	Fig. 3B and F and 4A
45	37	F	475	866	<50	43	110	AZT + 3TC + EFV	Fig. 3B and F and 4A
46	38	M	698	914	<50	32	66	TDF + 3TC + EFV	Fig. 3B and F and 4A
47	44	M	502	582	<50	26	47	TDF + 3TC + EFV	Fig. 3B and 4A
48	38	M	550	744	<50	20	139	AZT + 3TC + EFV	Fig. 3B and 4A
49	28	M	790	3,497	<50	70	81	ABC + 3TC + EFV	Fig. 3B and 4A
50	51	F	603	950	<50	109	103	AZT + 3TC + NVP	Fig. 4B
51	42	F	619	647	<50	1	39	TDF + 3TC + EFV	Fig. 4B
52	64	F	647	523	<50	112	163	TDF + 3TC + EFV	Fig. 4B
53	27	M	737	1,086	<50	6	59	AZT + 3TC + LPV/r	Fig. 4B
54	31	M	677	789	<50	25	71	TDF + 3TC + EFV	Fig. 4B
55	42	M	815	889	<50	18	101	AZT + 3TC + EFV	Fig. 4B
56	33	F	478	699	<50	20	100	d4T + 3TC + EFV	Fig. 4B
57	75	M	490	410	<50	30	76	TDF + 3TC + EFV	Fig. 4C
58	30	M	830	600	<50	15	63	TDF + 3TC + EFV	Fig. 4C
59	36	M	491	1,001	<50	49	90	TDF + 3TC + EFV	Fig. 4C
60	33	M	742	873	<50	26	72	TDF + 3TC + EFV	Fig. 4C
61	44	M	565	1,110	<50	9	45	TDF + 3TC + EFV	Fig. 4C
62	38	M	588	810	<50	11	76	AZT + 3TC + EFV	Fig. 4C
63	43	F	727	723	<50	2	88	AZT + 3TC + EFV	Fig. 4C
64	49	M	983	689	<50	70	133	AZT + 3TC + NVP	Fig. 4D and E; Table 3
65	51	F	586	921	<50	23	108	TDF + 3TC + EFV	Fig. 4D and E; Table 3
66	68	M	455	1,540	<50	3	120	d4T + 3TC + NVP	Fig. 4D and E; Table 3
67	43	M	1,570	960	<50	48	154	AZT + 3TC + EFV	Fig. 4D and E; Table 3
68	30	M	659	950	<50	2	84	AZT/3TC + NVP	Fig. 4D and E; Table 3
69	54	F	609	694	<50	43	72	TDF + 3TC + EFV	Fig. 4D and E; Table 3
70	42	F	556	652	<50	1	53	AZT + 3TC + EFV	Fig. 4D and E; Table 3
71	33	F	590	780	<50	18	51	d4T + 3TC + EFV	Fig. 4D and E; Table 3

a ND, not determined; t.n., treatment naive; ABC, abacavir; AZT, azidothymidine; EFV, efavirenz; d4T, stavudine; NVP, nevirapine; TDF, tenofovir disoproxil; 3TC, lamivudine; AZT/3TC, Combivir; LPV, lopinavir/r (boosted ritonavir; Kaletra); M, male; F, female.

**TABLE 2:** Clinical characteristics of the HIV-1-infected donors for which data are shown in Fig. 1B

Sample identifier	Age	Sex^*a*^	Cell count (no. of cells/μl)				
			Before ART		1 yr after ART		Viral load (no. of copies/ml)
			CD4^+^ T cells	CD8^+^ T cells	CD4^+^ T cells	CD8^+^ T cells	
6	27	M	107	685	344	659	<20
7	29	M	304	771	499	926	<20
1	23	M	321	822	514	750	<20
9	27	M	100	616	765	888	<20
10	55	M	267	1,267	195	906	51
11	27	M	280	518	332	1,420	<20
12	24	M	291	782	728	889	<20
4	24	F	250	834	370	616	<20
14	43	F	250	724	333	813	<20
15	39	M	65	658	434	1,601	<20
16	37	M	102	402	384	1,126	<20
17	49	M	45	308	135	583	<20
18	18	F	249	573	524	738	<20

a F, female; M, male.

10.1128/mBio.02121-19.1 FIG S1 Surface marker characterization of CD161^+^ CD4^+^ T cells from HIV-1-negative or HIV-1-infected donors. (A) Percentage of CD25-, CD69-, HLA-DR-, and CD38-expressing cells among peripheral CD161-positive or -negative CD4^+^ T lymphocytes from HIV-1-positive subjects receiving (*n* = 8) or not receiving (*n* = 17) ART or HIV-1-negative subjects (*n* = 12). (B) Frequency of cells expressing CXCR4 (left) and CCR5 (right) within bulk or CD45RO^+^ CD161-positive and -negative subsets from healthy donor PBMCs (*n* = 10) or lymph nodes (*n* = 6). Result are shown as the mean ± SEM for each group. Significant intergroup differences were determined using rank Kruskal-Wallis tests incorporating Dunn’s tests for multiple comparisons. Student’s *t* test was used to compare the statistical significance between cell subsets. *, *P < *0.05; **, *P < *0.01; ***, *P < *0.001. Download FIG S1, EPS file, 0.3 MB. Copyright © 2019 Li et al. 2019 Li et al. This content is distributed under the terms of the Creative Commons Attribution 4.0 International license.

10.1128/mBio.02121-19.3 FIG S3 CD161^+^ CD45RO^+^ CD4^+^ T cells are primarily of the memory phenotype with typical Th17 and pTFH characteristics. (A) Representative FACS plots showing CD45RA and CD45RO expression on CD161-positive (filled histogram) and -negative (open histogram) CD4^+^ T cells from healthy donor PBMCs. Cumulative data from several independent experiments show the CD45RA and CD45RO expression from healthy donor PBMCs (*n* = 14) or LN cells (*n* = 6) or ART-treated HIV-1-infected subject PBMCs (*n* = 10). (B) Percentage of CD4^+^ T cell subsets among CD161-positive and -negative cells in a cohort of 15 HIV-1-infected individuals with undetectable viral loads. (C) Dot plots comparing cytokine production among bulk or CD45RO^+^ CD161-positive or -negative CD4^+^ T cells from healthy donor PBMCs (*n* = 8) or LN cells (*n* = 5). (D) Expression of CXCR5 among bulk or CD45RO^+^ cell subsets from healthy donor PBMCs (*n* = 8) or LN cells (*n* = 5). (E) Statistical results of CCR5 expression in CCR6-positive or -negative CD45RO^+^ CD4^+^ T cell subsets in PBMCs from healthy donors (*n* = 8). (F and G) CD4^+^ T cells from healthy donor PBMCs were sorted, and then the cells were infected with R5-tropic strain NL4-3 (BaL *env*) for 5 days. The infectivity of different CD45RO^+^ CD4^+^ T cell subsets was assessed at 5 days after infection by flow cytometry (*n* = 6). Rank Kruskal-Wallis tests incorporating Dunn’s tests or Student’s *t* test was used for the analysis. The mean ± SEM for subsets from each group is shown. *, *P < *0.05; **, *P < *0.01; ***, *P < *0.001. Download FIG S3, EPS file, 0.6 MB. Copyright © 2019 Li et al. 2019 Li et al. This content is distributed under the terms of the Creative Commons Attribution 4.0 International license.

To determine the susceptibility of CD161^+^ CD4^+^ T cells to HIV-1 infection, we first quantified the expression level of the coreceptors CCR5 and CXCR4 from HIV-1-negative and -positive individuals receiving or not receiving ART. CXCR4 was expressed at high and similar levels on these two T cell subsets in the three groups (Fig. 1D) and also in the memory subsets of CD161-positive and -negative CD4^+^ T cells from healthy donor peripheral blood mononuclear cells (PBMCs) and lymph nodes (Fig. S1B). However, CCR5 expression was significantly higher on CD161^+^ CD4^+^ T cells than on CD161-negative cells both in healthy individuals and in HIV-1-infected donors (Fig. 1D). Memory CD161^+^ CD4^+^ T cells from HIV-1-negative PBMCs and lymph nodes also expressed higher levels of CCR5 than CD161^−^ CD4^+^ T cells (Fig. S1B). We further investigated the susceptibility of these two T cell subsets to R5- or X4-tropic HIV-1 infection *in vitro*. Regardless of whether the cells were prestimulated with anti-CD3 and anti-CD28 antibodies or not, infectivity in CD161^+^ CD4^+^ T cells was significantly higher than that in CD161^−^ CD4^+^ T cells at various time points postinfection (Fig. 1E and F), and this was also the case for CD161-positive and -negative CD45RO^+^ CD4^+^ T cells from healthy donor PBMCs (Fig. S2). Taken together, our data clearly indicate that CD161^+^ CD4^+^ T cells are highly permissive for HIV-1 infection and that the reduced amount of these cells in infected individuals may be due to the death of the infected cells.

10.1128/mBio.02121-19.2 FIG S2 CD161 ^+^ CD45RO^+^ CD4^+^ T cells are highly permissive for HIV-1 infection. CD45RO^+^ CD4^+^ T cell subsets from HIV-negative subjects were sorted and prestimulated with anti-CD3/CD28 for 3 days and then exposed to NL4-3 (*n* = 5; left) or NL4-3 (BaL *env*) (*n* = 5; right). Intracellular HIV-1 p24 expression was quantified by FACS. Download FIG S2, EPS file, 0.2 MB. Copyright © 2019 Li et al. 2019 Li et al. This content is distributed under the terms of the Creative Commons Attribution 4.0 International license.

### CD161^+^ CD4^+^ T cells are primarily of the memory phenotype with typical of Th17 and pTFH characteristics.

**FIG 2:**
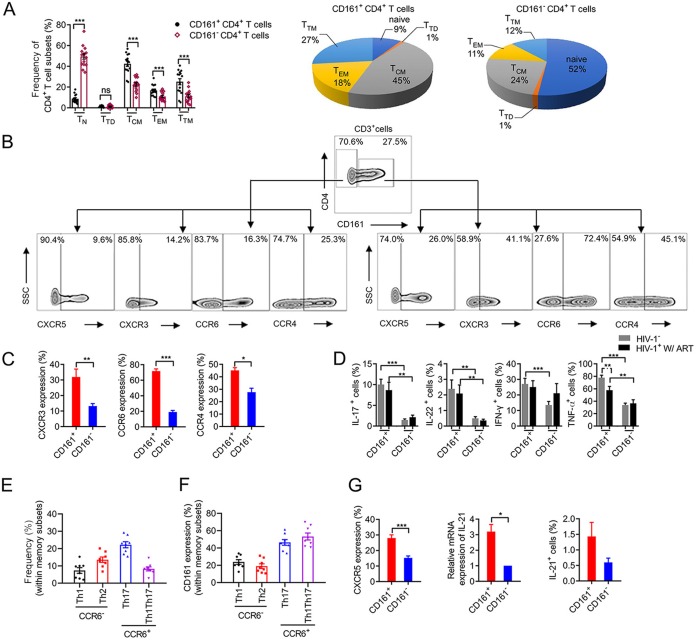
CD161^+^ CD4^+^ T cells are primarily of the memory phenotype with typical Th17 and pTFH characteristics. (A) Percentage of CD161-positive and -negative cells in different CD4^+^ T cell subsets and composition of CD161-positive and -negative CD4^+^ T cells among healthy individuals (*n* = 15). T_N_, naive T cells; T_TD_, terminally differentiated T cells; T_CM_, central memory T cells; T_EM_, effector memory T cells; T_TM_, transitional memory T cells. (B and C) Representative FACS plots depicting the chemokine receptor profile, including CXCR5, CCR6, CCR4, and CXCR3 expression, on CD161^−^ CD4^+^ (left) and CD161^+^ CD4^+^ (right) T cells (B). Cumulative results from 6 independent experiments are shown in panel C. SSC, side scatter. (D) Cytokine production profile. PBMCs from healthy donors (*n* = 14) or HIV-1-infected subjects receiving ART for more than 2 years (*n* = 8) were stimulated with phorbol-12-myristate-13-acetate and ionomycin in the presence of brefeldin A for 8 h. Dot plots comparing cytokine production among CD161-positive or -negative CD4^+^ T cells. IFN-γ, gamma interferon, TNF-a, tumor necrosis factor alpha. (E and F) Frequency and CD161 expression levels of Th1, Th2, Th17, and Th1Th17 cells. CD45RO^+^ CD4^+^ T cells from healthy donors (*n* = 8) were analyzed for their diﬀerential expression of CCR6, CCR4, and CXCR3. The CCR6^+^ subsets included the CCR4^+^ CXCR3^−^ (Th17) and CCR4^−^ CXCR3^+^ (Th1Th17) subsets. The CCR6^−^ subsets included the CCR4^+^ CXCR3^−^ (Th2) and CCR4^−^ CXCR3^+^ (Th1) subsets. (G) CXCR5 and IL-21 expression in CD161-positive and -negative cells. CXCR5 expression was measured by FACS. For IL-21 measurement, FACS-purified CD161-positive or -negative CD4^+^ T cells from healthy donors were stimulated by anti-CD3/CD28 for 3 days, and the IL-21 mRNA level (*n* = 4) was measured by RT-qPCR as the level of expression relative to that for GADPH. IL-21 (*n* = 6) secretion was analyzed by intracellular staining as described in the legend to D. *P* values were determined by the Mann-Whitney test or Student's *t* test. The mean ± SEM is shown. *, *P < *0.05; **, *P < *0.01; ***, *P < *0.001.

The HIV-1 latent reservoir has been shown to be composed of mainly resting memory CD4^+^ T cells (5, 27). We characterized the phenotype of CD161^+^ CD4^+^ T cells to determine if it was in line with the features of latently infected cells. Roughly 90% of CD161^+^ CD4^+^ T cells expressed CD45RO but not CD45RA (Fig. S3A). Using CCR7 and CD27 expression to further distinguish memory subsets, we found that nearly 50% of CD161^+^ CD4^+^ T cells were of the central memory T cell (T_CM_) phenotype, with slightly fewer cells exhibiting the transitional memory T cell (T_TM_) and effector memory T cell (T_EM_) phenotypes (Fig. 2A). On the contrary, the CD161-negative population was primarily of the naive phenotype, with only 20% of it being T_CM_ cells (Fig. 2A). We also found that the composition of memory cell subsets of CD161^+^ cells in HIV-1-infected donors under ART was similar to that in healthy individuals (Fig. S3B). To gain insight into the lineage commitment and homing properties of CD161^+^ CD4^+^ T cells, we examined their chemokine receptor profile. CCR6 was expressed on most (72%) CD161-positive CD4^+^ T cells, in contrast to the findings for the CD161-negative subsets (19%) (Fig. 2B and C). CD161^+^ CD4^+^ T cells from HIV-1-infected and healthy donor PBMCs produced larger amounts of IL-17A, IL-22, gamma interferon, and tumor necrosis factor alpha than CD161^−^ CD4^+^ T cells after *ex vivo* stimulation with phorbol-12-myristate-13-acetate (PMA) and ionomycin (Fig. 2D), as did the memory subset of CD161^−^ CD4^+^ T cells (Fig. S3C). CD161^+^ CD4^+^ T cells from healthy donor LN cells also secreted more IL-17A and IL-22 than CD161^−^ CD4^+^ T cells (Fig. S3C). To further investigate the expression of CD161 in different T helper cell subsets, we used CCR4, CXCR3, CCR6, and CD45RO to identify Th1, Th2, Th17, and Th1Th17 cells. The frequency of Th17 and Th2 cells was higher among CCR6-positive and -negative cells, respectively. The expression of CD161 was higher in Th17 and Th1Th17 cells than in Th1 or Th2 cells (Fig. 2E and F). Peripheral follicular T helper (pTFH) cells have recently been shown to be a major viral replication cellular compartment and harbor a significant amount of intracellular HIV-1 proviral DNA (28). We found that CD161^+^ CD4^+^ T cells expressed higher levels of CXCR5 than CD161^−^ CD4^+^ T cells both in blood and in LN from healthy donors (Fig. 2G; Fig. S3D). The production of IL-21 was also significantly higher in CD161^+^ CD4^+^ T cells than in CD161^−^ CD4^+^ T cells after being stimulated *in vitro* at the RNA and protein levels (Fig. 2G).

To further confirm whether the CD161^+^ CD4^+^ T cells among the Th17 and Th1Th17 cell subsets were highly permissive to HIV-1 infection, we performed an *in vitro* infectivity assay. Our results showed that CCR5 expression was higher on CD161-positive cells than on CD161-negative cells for the Th1, Th2, and Th1Th17 subsets (Fig. S3E). The Th1Th17 subset was more permissive to R5-tropic HIV-1 than the other subsets (Fig. S3F). Finally, the infectivity of CD161^+^ cells was higher than that of CD161^−^ cells in these four cell populations (Fig. S3G). Together, our data demonstrated that CD161^+^ CD4^+^ T cells are primarily of the memory phenotype with typical characteristics of Th17 and pTFH cells; thus, they possess the potential to be latently infected with HIV-1.

### CD161^+^ CD4^+^ T cells exhibit higher survival and proliferative abilities than CD161^−^ CD4^+^ T cells.

**FIG 3:**
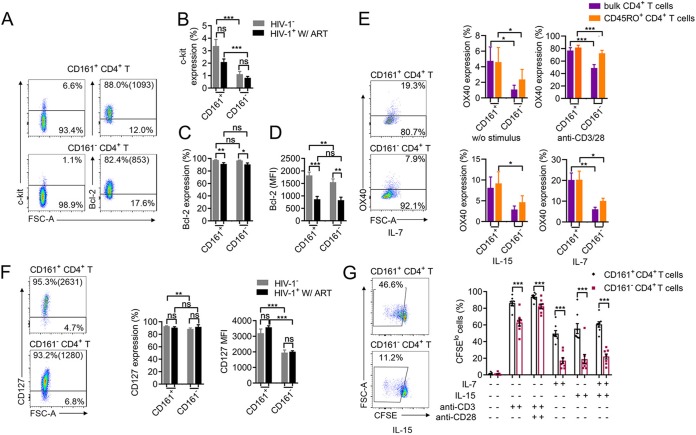
CD161^+^ CD4^+^ T cells have higher survival and proliferative abilities than CD161^−^ CD4^+^ T cells. (A) Representative FACS plots showing c-kit and Bcl-2 expression. (B) c-kit expression on CD161-positive and -negative CD4^+^ T cells from healthy donors (*n* = 17) or ART-treated HIV-1-infected subjects (*n* = 7). (C and D) The percentage (C) and mean fluorescence intensity (D) of Bcl-2 expression by cells from 8 healthy individuals or 9 ART-treated HIV-1-infected donors. (E) OX40 expression in bulk or memory CD4^+^ T cells under different stimuli. PBMCs from healthy donors (*n* = 6) were treated without a stimulus or with anti-CD3/CD28 for 3 days and IL-7 or IL-15 for 6 days, and OX40 expression by bulk or memory CD4^+^ T cell subsets was measured. (F) IL-7 receptor (CD127) expression on CD4^+^ T cell subsets. Results are for healthy donors (*n* = 13) and HIV-1-infected individuals under ART (*n* = 4). (G) Proliferation of CD161-positive and -negative CD4^+^ T cells under different stimuli *in vitro*. PBMCs from healthy donors (*n* = 8) were loaded with carboxyfluorescein succinimidyl ester (CFSE) and cultured for 6 days with the indicated stimulus. The percentages of CFSE^lo^ cells among CD161-positive and -negative CD4^+^ T cells are shown. The data represent the mean ± SEM. *P* values were determined by the Mann-Whitney U test or Student’s *t* test. ns, not significant; *, *P < *0.05; **, *P < *0.01; ***, *P < *0.001.

Having demonstrated that CD161^+^ CD4^+^ T cells are highly permissive to HIV-1 infection, we went on to further investigate whether these cells have the potential to survive longer and proliferate *in vitro*, which is a critical prerequisite for promoting the clonal expansion of latent HIV-1. c-kit and Bcl-2 are key signaling molecules for cellular survival (29, 30). We found that the percentage of c-kit expression was significantly higher on CD161^+^ CD4^+^ T cells than on the negative populations in both healthy and infected individuals. The mean fluorescence intensity (MFI) of Bcl-2 was also higher on CD161-positive CD4^+^ T cells than CD161-negative CD4^+^ T cells from healthy donors (Fig. 3A to D). This pattern remained the same in the memory subsets of CD161-positive and -negative CD4^+^ T cells from healthy donors (Fig. S4A). OX40 was previously shown to be associated with the long-term survival and the clonal proliferation of CD4^+^ T cells and may denote cells harboring a higher frequency of latent HIV-1 (31, 32). Here, we found that either without stimulus or with anti-CD3/CD28, IL-15, or IL-7 treatment, both bulk and memory CD161^+^ CD4^+^ T cells expressed higher levels of OX40 than their CD161-negative counterparts (Fig. 3E). IL-7 or IL-15 is an important stimulus for the homeostatic proliferation of memory CD4^+^ T cells (33–35). To explore the potential to receive IL-7 signaling, we measured IL-7 receptor (CD127) expression on CD161-positive and -negative CD4^+^ T cells. Although the percentage of cells expressing CD127 was comparable between the two groups, the MFI of CD127 was significantly higher on CD161^+^ CD4^+^ T cells than on CD161^−^ CD4^+^ T cells from both healthy and infected individuals (Fig. 3F). This pattern remained the same with the memory subsets of CD161-positive and -negative CD4^+^ T cells (Fig. S4B). We further examined the proliferative ability of CD161^+^ CD4^+^ T cells in response to *in vitro* anti-CD3/CD28, IL-15, or IL-7 treatment. Compared with their CD161-negative counterparts, CD161^+^ CD4^+^ T cells proliferated significantly better with TCR or homeostatic cytokine signals (Fig. 3G). Even within the memory subset, CD161^+^ CD4^+^ T cells showed a higher proliferative ability than CD161^−^ CD4^+^ T cells under the same treatment conditions with or without IL-2 (Fig. S4C). Taken together, CD161^+^ CD4^+^ T cells possess characteristics of long-lived memory T cells and undergo significant proliferation in response to *in vitro* antigenic or homeostatic signaling, suggesting that these cells have the potential to promote the clonal expansion of latent HIV-1 if they become infected.

10.1128/mBio.02121-19.4 FIG S4 CD45RO^+^ CD161^+^ CD4^+^ T cells from healthy donors have higher survival and proliferative abilities. (A) Expression level of c-kit or Bcl-2 in bulk or CD45RO ^+^ CD161-positive or -negative CD4^+^ T cells from healthy individuals. (B) CD127 expression level in CD161-positive and -negative cells within CD45RO^+^ or bulk CD4^+^ T cells. (C) Proliferation of CD161-positive and -negative CD45RO^+^ or CD45RO^−^ CD4^+^ T cells under different stimuli in vitro. PBMCs form healthy donors (*n* = 6) were CFSE loaded and cultured for 6 days with anti-CD3 MAbs, anti-CD3 and anti-CD28 MAbs, IL-7, IL-15, or IL-7/IL-15 in the presence or absence of IL-2. The percentages of CFSE^lo^ cells among CD161-positive and -negative CD45RO^+^ CD4^+^ T cells are shown. Data represent the mean ± SEM. *P* values are from Student’s t test. ns, not significant; *, *P < *0.05; **, *P < *0.01; ***, *P < *0.001. Download FIG S4, EPS file, 0.4 MB. Copyright © 2019 Li et al. 2019 Li et al. This content is distributed under the terms of the Creative Commons Attribution 4.0 International license.

### CD161^+^ CD4^+^ T cells harbor more replication-competent latent HIV-1 and clonal expanded proviruses than CD161^−^ CD4^+^ T cells.

**FIG 4:**
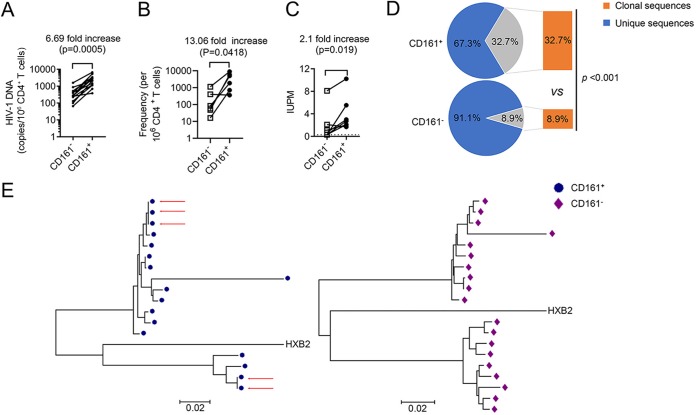
CD161^+^ CD4^+^ T cells harbor more replication-competent latent HIV-1 and clonal expanded proviruses than CD161^−^ CD4^+^ T cells. (A) Frequency of HIV-1 total proviral DNA in CD161-positive and -negative CD4^+^ T cells from ART-suppressed infected individuals (*n* = 13) measured by qPCR. (B) IPDA results for CD161-positive and -negative CD4^+^ T cells from ART-suppressed infected individuals (*n *=* *6). (C) Frequency of replication-competent latent HIV-1 in CD161-positive and -negative CD4^+^ T cells measured by QVOA. IUPM, infectious units per million cells. A ratio-paired *t* test was performed for statistical analysis. (D) Clonality of the proviral *env* sequence recovered from CD161-positive or -negative CD4^+^ T cells within the total number of sequences. Significance was calculated using a chi-square test; the *P* value is indicated. (E) Representative neighbor-joining phylogenetic tree constructed with *env* sequences recovered from CD161-positive or -negative CD4^+^ T cells by single-genome proviral sequencing. Red arrows, identical clonal expansion sequences; blue symbols, sequences detected in CD161-positive CD4^+^ T cells; purple symbols, sequences detected in CD161-negative CD4^+^ T cells. See Table 3 for patient characteristics.

If infected CD161^+^ CD4^+^ T cells indeed promote the expansion of the HIV-1 latent reservoir, we should see a higher frequency of latent HIV-1 in these cells. Here, we used a quantitative PCR (qPCR) assay, an intact proviral DNA assay (IPDA) (36), and a quantitative viral outgrowth assay (QVOA) (37) to measure the reservoir size in purified CD161-positive and -negative CD4^+^ T cells from ART-suppressed infected individuals. The data clearly showed that the frequencies of both proviral DNA and replication-competent latent HIV-1 were significantly higher in CD161^+^ CD4^+^ T cells than in CD161^−^ CD4^+^ T cells by the three different approaches, with 6.7-fold, 13.0-fold, and 2.1-fold enrichment in the positive population, respectively (Fig. 4A to C). HIV-1 latently infected cells possess a remarkable stability and persist long term in HIV-1-infected subjects despite successful ART (4, 38, 39). The mechanism of memory cell homeostasis suggests that the stability of the latent reservoir could be partially dependent upon the ability of infected cells to proliferate. Phylogenetic analysis established that identical proviral sequences in HIV-1-infected individuals under ART can reflect expanded cellular clones (40). Each set of identical *env* sequences is most likely to represent a clonal population of infected cells derived from a single cell initially infected by HIV-1 (10).

To further evaluate whether the cellular proliferation of CD161^+^ CD4^+^ T cells can act as an important driving force for HIV-1 persistence, we analyzed the proviral *env* sequence by limiting dilution PCR and single-genome proviral sequencing. We recovered identical proviral *env* sequences from 6 out of 8 subjects analyzed. The CD161^+^ CD4^+^ T cells in all 6 subjects harbored identical proviral *env* sequences, whereas the CD161^−^ CD4^+^ T cells from only 3 subjects harbored clonal expanded sequences. A total of 32.7% of the proviral *env* sequences recovered from CD161^+^ CD4^+^ T cells but only 8.9% of those from their CD161-negative counterparts were clonal (Fig. 4D). A summary of the results of phylogenetic analyses of proviral HIV-1 *env* sequences is shown in Table 3. A representative tree of one infected individual is shown in Fig. 4E, and the results for the other donors are shown in Fig. S5. Together, this direct evidence from ART-treated individuals strongly indicates that HIV-1-infected CD161^+^ CD4^+^ T cells undergo clonal expansion *in vivo*, therefore composing a significantly larger HIV-1 latent reservoir in these cells than in other cells.

**TABLE 3:** Number of sequences obtained from each subset and participant

Participant identifier	CD161^+^ CD4^+^ T cells				CD161^−^ CD4^+^ T cells			
	No. of sequences analyzed	No. of clones found	No. of sequences from each clone	Clone rate (%)	No. of sequences analyzed	No. of clones found	No. of sequence from each clone	Clone rate (%)
1	17	2	2, 3	29.4	19	0	0	0.0
2	30	1	4	13.3	28	1	2	7.1
3	20	1	2	10.0	21	0	0	0.0
4	38	2	2, 24	68.4	22	0	0	0.0
5	15	0	0	0.0	13	0	0	0.0
6	11	0	0	0.0	10	0	0	0.0
7	10	2	2, 7	90.0	10	2	3, 3	60.0
8	18	3	2, 2, 2	33.3	12	1	4	33.3
								
Total	159	11	52	32.7	135	4	12	8.9

10.1128/mBio.02121-19.5 FIG S5 CD161^+^ CD4^+^ T cells harbor more HIV-1 clonal expanded proviruses than CD161^−^ CD4^+^ T cells. Neighbor-joining phylogenetic tree constructed with HIV-1 *env* sequences obtained from CD161-positive and -negative CD4^+^ T cells from 7 HIV-1-infected study subjects on ART with undetectable plasma viral loads and CD4 cell counts above 450 per mm^3^. Red arrows, identical clonal expanded sequences; blue symbols, sequences detected in CD161^+^ CD4^+^ T cells; purple symbols, sequences detected in CD161^−^ CD4^+^ T cells. Download FIG S5, EPS file, 0.7 MB. Copyright © 2019 Li et al. 2019 Li et al. This content is distributed under the terms of the Creative Commons Attribution 4.0 International license.

## DISCUSSION

Previous works showed that the distribution of latently infected CD4^+^ T cells across different subsets is not equal (41). For example, central memory T cells (11), Th17 cells (13), and follicular T helper cells (42) largely contribute to viral persistence. The documented role of CD161^+^ CD4^+^ T cells in pathogenic autoimmunity (17, 43) and their lineage connection to Th17 cells make them a possible candidate to serve as HIV-1 targets at the portal of entry (44). We observed that CD161^+^ CD4^+^ T cells were highly permissive to X4- or R5-tropic HIV-1 infection and harbored total HIV-1 DNA at significantly higher frequencies than the CD161-negative population. IPDA and QVOA further demonstrated that CD161^+^ CD4^+^ T cells make up a substantial portion of the replication-competent HIV-1 latent reservoir. Latently infected T cells that persist under successful ART do not express viral antigens, making it nearly impossible to target these cells for clearance (41). The identification of viable markers of latently infected cells is therefore urgent. Some molecules, such as immune checkpoint inhibitors (45), CD32a (46), or CD30 (47), have recently been reported to be markers of the latent reservoir, but the use of these as markers is still under debate. It has also been reported that CCR5-expressing cells enrich latently infected cells, but CCR5 is not an ideal marker because its expression turns on and off (48). Our experiments showed that CD161 expression on CD4^+^ T cells is relatively stable, and when this characteristic is combined with the fact that latent HIV-1 is highly enriched in these cells, CD161 is a promising marker that may help to identify latently infected cells and a potential marker for targeting the latent reservoir.

A series of recent studies has indicated that the clonal proliferation of infected T cells carrying replication-competent virus is a major mechanism underlying the long-term stability of the HIV-1 latent reservoir (7, 10, 49, 50). It was reported that functionally polarized Th1 CD4^+^ T cells carry more clonally expanded genome-intact HIV-1 (7). Th17 cells were shown to possess the characteristics of proliferative self-renewal, survival, and apoptotic resistance (21). Besides, a recent study supported the concept that HIV-1 takes advantage of the long-lived properties of specific Th17 cell subsets to ensure its persistence under ART treatment (13). According to previously recognized hallmarks of Th17 cells, analyses of such Th17 clones revealed the specific expression of CD161 (15). IL-17-producing cells differentiate from CD161 precursors and maintain the expression of CD161 throughout their life cycle. The C-type lectin-like receptor CD161, which has been described to promote T cell expansion (24), is expressed on a discrete subset of human CD4^+^ T cells (22). Our results showed that CD161^+^ CD4^+^ T cells had higher rates of survival and a higher proliferative ability than their CD161-negative counterparts with TCR or homeostatic signals. Considering the enrichment of replication-competent latent HIV-1 and clonal expanded proviral sequences, CD161^+^ CD4^+^ T cells may act as a major cellular subset that drives clonal expansion.

Computational analysis predicted that the inhibition of infected CD4^+^ T cell proliferation may decrease the half-life of latently infected cells by up to 20-fold (51). Therefore, perturbation of the clonal expansion of latently infected cells or the CD161^+^ CD4^+^ T cell population may provide a promising strategy to counterbalance the repopulation and dissemination of latently infected cells and reduce the size of the latent reservoir, as we have demonstrated here. On the other hand, the application of latency-reversing agents (LRAs) needs to be revisited, since some LRAs, like IL-15 or IL-15 agonist, could actually promote the expansion of the HIV-1 latent reservoir, and we reported this effect on CD161^+^ CD4^+^ T cells here. Further evidence is still needed to understand the intrinsic cellular program by which these cells maintain self-renewal and survival. Pharmaceutical inhibition of CD161^+^ CD4^+^ T cells or specific signaling pathways needs to be investigated for its ability to restrict the clonal proliferation of infected cells, which may be translated into improved clinical strategies for HIV-1 functional cure and eradication.

## MATERIALS AND METHODS

### Study subjects.

Peripheral blood for the isolation of PBMCs or CD4^+^ T cells was obtained from 71 HIV-1-infected patients and 20 healthy adult participants. Among them, 53 patients had been on antiretroviral therapy (ART) for at least 2 years and had maintained undetectable plasma HIV-1 RNA levels (<50 copies per ml) for at least 1 year before blood collection, and their CD4^+^ counts were more than 450 cells per μl. In addition, 18 patients who had recently been diagnosed with HIV-1 infection but who had not started treatment were recruited and followed for 1 year. This study was approved by the Ethics Review Boards of Sun Yat-sen University and the Eighth People’s Hospital of Guangzhou, Guangzhou, China. HIV-1-infected patients were recruited at The Eighth People’s Hospital, and all were written consent informed. Normal human lymph node tissue obtained during exploration of the abdominal cavity in patients with nonneoplastic acute abdomen at Guangzhou First People’s Hospital was used for the cytoimmunofluorescence staining technique under a study protocol approved by the Institutional Review Board of Guangzhou First People's Hospital. The use of PBMCs from healthy adult donors was approved by the Institutional Review Board of Guangzhou Blood Center. The age of the HIV-negative donors included in our study ranged from 18 to 65 years. CD4 and CD8 counts were in the normal range of clinical test values, and none of the donors was infected with HIV-1.

### Isolation and culture of primary human T lymphocytes.

PBMCs derived from HIV-1-infected patients or healthy donors were isolated by Ficoll-Paque gradient separation. Primary CD4^+^ T cells were obtained from PBMCs by negative magnetic selection with human CD4^+^ T lymphocyte enrichment set DM (BD IMag cell separation system). Then, CD161-positive or -negative and bulk CD4^+^ T cells were sorted by use of a FACSAria cell sorter (BD Biosciences) upon staining with CD3, CD4, and CD161 surface-staining antibodies. Cells were sorted and resulted in the isolation of lymphocytes with the defined phenotypic characteristic of >95% purity. Data were analyzed using FlowJo software (Tree Star Inc., Ashland, OR, USA). The isolated T cells were cultured in basal medium or were stimulated for 3 days with anti-CD3 antibody at 1 μg ml^−1^ (BioLegend) or anti-CD28 antibody at 1 μg ml^−1^ (BioLegend) before wild-type HIV-1 infection. CD4^+^ T cells or PBMCs were stimulated with anti-CD3 (1 μg ml^−1^) or anti-CD3 and anti-CD28 (1 μg ml^−1^) monoclonal antibodies (MAbs), IL-7 (10 ng ml^−1^), and IL-15 (10 ng ml^−1^) or with IL-7 and IL-15 only with or without IL-2 (100 U ml^−1^). All cell culture basal media contained 90% RPMI 1640 supplemented with 10% fetal bovine serum, 100 U ml^−1^ penicillin, and 100 μg ml^−1^ streptomycin (Gibco, Invitrogen, Carlsbad, CA), and cell cultures were maintained in an environment of 37°C and 5% CO_2_.

### Intracellular cytokine or HIV-1 p24 staining.

To assess intracellular staining for cytokines, PBMCs stimulated with PMA (50 ng ml^−1^)-ionomycin (1 μg ml^−1^) (8 h) and brefeldin A (10 μg ml^−1^) were added at the last 5 h of stimulation to inhibit cytokine release.

Cells were stained with monoclonal antibodies to the surface markers for 30 min on ice in the dark and washed with wash medium twice and then fixed by the use of fixation/permeabilization buffer (BD Biosciences), followed by intracellular cytokine or HIV-1 p24 staining with antibodies directed against intracellular antigen. Details of the antibodies used for flow cytometry are provided in Table S1 in the supplemental material. Cells were analyzed by fluorescence-activated cell sorting (FACS) using a BD LSR Fortessa flow cytometer and FlowJo software (Tree Star Inc., Ashland, OR, USA).

10.1128/mBio.02121-19.6 TABLE S1 Fluorochrome-conjugated antibodies used in flow cytometry. Download Table S1, DOCX file, 0.02 MB. Copyright © 2019 Li et al. 2019 Li et al. This content is distributed under the terms of the Creative Commons Attribution 4.0 International license.

### Virus production and *in vitro* HIV-1 infection.

On the day before transduction, HEK293T cells were seeded at 8 × 10^6^ cells per 100-mm dish. Twenty-four hours later, virus was generated by transfecting HEK293T cells with a plasmid encoding NL4-3 or NL4-3 BaL, using a polyethylenimine transfection system and following the manufacturer’s instructions. Supernatants were harvested after 48 h, centrifuged (10 min, 500 × *g*, room temperature), and filtered through a 0.45-μm-pore-size membrane to remove the cell debris. Viruses were concentrated by centrifuging with a 25% volume of 50% polyethylene glycol 6000 and a 10% volume of 4 M NaCl. Concentrated virions were resuspended in complete medium and stored at −80°C. The virus concentration was estimated by p24 titration using an enzyme-linked immunosorbent assay (ELISA). Different CD4^+^ T cell subsets cultured in basal medium or stimulated for 3 days with anti-CD3 antibody at 1 μg ml^−1^ (BioLegend) and anti-CD28 antibody at 1 μg ml^−1^ (BioLegend) were infected with NL4-3 Bal or NL4-3 virus (p24 titer, 50 ng ml^−1^). Infected CD4^+^ T cells were further cultured in basal medium and incubated at 37°C in a humidified incubator with 5% CO_2_. Then, infected cells were analyzed by flow cytometry at 3 to 7 days postinfection.

### Assessment of HIV-1 proviral DNA and replication-competent HIV-1 in patient primary CD161-positive or -negative CD4^+^ T cells.

To compare the frequency of proviral DNA, two subsets of cells, including CD161-positive and -negative CD4^+^ T cells, were purified by FACS. Sorted CD4^+^ T cell populations were subjected to DNA and RNA extraction using commercial kits purchased from Magen (catalog number R5111-02). Genomic DNA was collected for quantitative PCR using Gag primers and probe, which are described in Table S2. qPCR was performed using the following program: 50°C for 2 min, 95°C for 10 min, and 40 cycles of 94°C for 15 s and 60°C for 1 min. An intact proviral DNA assay (IPDA), which is a quantitative approach for measuring the reservoir of latent HIV-1 proviruses, was used to measure the frequency of latent HIV-1 proviral DNA between CD161-positive and -negative cells (36). Briefly, genomic DNA from these two types of T cells was detected by droplet digital PCR (ddPCR) using a primer/probe mix consisting of oligonucleotides for two independent hydrolysis probe reactions that interrogate conserved regions of the HIV-1 genome to discriminate intact from defective proviruses, The ddPCR was performed on a Bio-Rad QX200 AutoDG digital droplet PCR system (Bio-Rad Laboratories). Simultaneous quantification of DNA shearing and input human genome equivalents was performed using another aliquot of the same DNA sample and two independent hydrolysis probe reactions that interrogate the human *RPP30* gene (Table S3). The number of proviral copies per 10^6^ CD4^+^ T cells can be estimated by using the DNA shearing index to correct the raw ddPCR output for *RPP30* and HIV-1. To measure the frequency of latent HIV-1, CD161-positive and -negative CD4^+^ T cells were obtained by FACS and then used for a limiting dilution virus outgrowth assay (37). Briefly, these two T cell subsets were cultured with irradiated PBMCs in the presence of phytohemagglutinin (PHA) and IL-2. The PHA was removed 18 h later. Fresh CD8-negative PBMCs from healthy donors were added to the culture on day 1 and day 7. The culture supernatant was collected at 14 and 21 days for the HIV-1 p24 ELISA.

10.1128/mBio.02121-19.7 TABLE S2 Gene-specific primers and probes. Download Table S2, DOCX file, 0.01 MB. Copyright © 2019 Li et al. 2019 Li et al. This content is distributed under the terms of the Creative Commons Attribution 4.0 International license.

10.1128/mBio.02121-19.8 TABLE S3 Gene-specific primers and probes used for IPDA. Download Table S3, DOCX file, 0.01 MB. Copyright © 2019 Li et al. 2019 Li et al. This content is distributed under the terms of the Creative Commons Attribution 4.0 International license.

### Sequencing of the *env* sequence and construction of phylogenetic trees.

Genomic DNA was extracted from the cell populations indicated above using a tissue DNA kit (Mega). The DNA was serially diluted 1:3, 1:9, 1:27, and 1:81, and for each dilution, 20 reactions with two rounds of nested PCR were performed using Invitrogen Platinum *Taq* high-fidelity polymerase and primers specific to the HIV-1 *env* regions. Briefly, for the first round of PCR, 2 μl diluted DNA was amplified in a 40-μl reaction mixture containing 1 μM of the primers indicated in Table S2, 1× high-fidelity buffer [180 mM (NH_4_)_2_SO_4_, 2 mM MgSO_4_, 600 mM Tris-SO_4_, pH 8.9, 0.2 mM deoxynucleoside triphosphates; Invitrogen), and 0.025 U/ml Platinum *Taq* high-fidelity polymerase (Invitrogen) (52). PCR conditions for the first round were 94°C for 2 min and then 94°C for 30 s, 60°C for 1 min, and 68°C for 5 min for 3 cycles; 94°C for 15 s, 60°C for 30 s, and 68°C for 4.5 min for 32 cycles; and then 68°C for 10 min. The first-round PCR product was diluted 1:3 in Tris-HCl (5 mM, pH 8), and 2 μl of the diluted reaction mixture was transferred to the 30-μl second-round reaction mixture using the primers listed in Table S2. Second-round PCR conditions were 94°C for 2 min; then 94°C for 15 s, 55°C for 30 s, and 68°C for 3 min for 35 cycles; and then 68°C for 10 min. Wells positive for amplified HIV-1 proviruses were identified by diluting the second-round PCR product 1:3 with Tris-HCl, followed by visualization on a 1% agarose gel. According to the Poisson distribution, the dilution at which 30% of the PCRs were positive has an 80% probability of containing a single amplified provirus. Therefore, the dilution at which approximately 30% of the amplicons were positive was selected, and additional PCRs were completed until all the DNA sample from two T cell subsets was amplified. The PCR products were then purified and either sequenced by regular Sanger sequencing or deep sequenced by use of an Illumina MiSeq platform. Neighbor-joining distance analysis was performed in MEGA (version 7) software. The average pairwise distances (APDs) were calculated in MEGA (version 7) software from *env* proviral single-genome sequences.

### Real-time RT-qPCR analysis.

Total RNA was isolated with the TRIzol reagent (Life Technologies) and then subjected to cDNA synthesis using an EasyScript One-Step genomic DNA removal and cDNA synthesis supermix kit (TransGen Biotech). Primers were obtained from PrimerBank and are shown in Table S2. The primers were annealed at 37°C, and reverse transcriptase (RT) was processed at 42°C. Quantitative PCR was performed with ChaQ SYBR qPCR master mix (Vazyme Biotech) by following the manufacturer’s instructions. Quantification was performed by normalization of the amount to that of the glyceraldehyde-3-phosphate dehydrogenase (GAPDH) housekeeping gene.

### Statistical analysis.

Data are presented as the mean ± standard error of the mean (SEM) from at least 4 independent experiments unless indicated otherwise in the figure legends. A Student's *t* test (paired) was applied to normally distributed data. One-way analysis of variance followed by Bonferroni’s correction (when two groups were compared), Dunnett’s test (when all experimental groups were compared to one control group), or Tukey’s multiple-comparison test (when all groups were compared to each other) was applied in the multiple comparisons after one-way analysis of variance (one-way ANOVA). Statistical significance was accepted at a *P *value of <0.05. Statistical analyses were performed using GraphPad Prism (version 7) software.

## References

[1] Gulick RM, Mellors JW, Havlir D, Eron JJ, Gonzalez C, McMahon D, Richman DD, Valentine FT, Jonas L, Meibohm A, Emini EA, Chodakewitz JA 1997 Treatment with indinavir, zidovudine, and lamivudine in adults with human immunodeficiency virus infection and prior antiretroviral therapy. N Engl J Med 337:734–739. doi:10.1056/NEJM199709113371102. 9287228

[2] Perelson AS, Essunger P, Cao Y, Vesanen M, Hurley A, Saksela K, Markowitz M, Ho DD 1997 Decay characteristics of HIV-1-infected compartments during combination therapy. Nature 387:188–191. doi:10.1038/387188a0. 9144290

[3] Davey RT Jr, Bhat N, Yoder C, Chun TW, Metcalf JA, Dewar R, Natarajan V, Lempicki RA, Adelsberger JW, Miller KD, Kovacs JA, Polis MA, Walker RE, Falloon J, Masur H, Gee D, Baseler M, Dimitrov DS, Fauci AS, Lane HC 1999 HIV-1 and T cell dynamics after interruption of highly active antiretroviral therapy (HAART) in patients with a history of sustained viral suppression. Proc Natl Acad Sci U S A 96:15109–15114. doi:10.1073/pnas.96.26.15109. 10611346 PMC24781

[4] Siliciano JD, Kajdas J, Finzi D, Quinn TC, Chadwick K, Margolick JB, Kovacs C, Gange SJ, Siliciano RF 2003 Long-term follow-up studies confirm the stability of the latent reservoir for HIV-1 in resting CD4^+^ T cells. Nat Med 9:727–728. doi:10.1038/nm880. 12754504

[5] Finzi D, Hermankova M, Pierson T, Carruth LM, Buck C, Chaisson RE, Quinn TC, Chadwick K, Margolick J, Brookmeyer R, Gallant J, Markowitz M, Ho DD, Richman DD, Siliciano RF 1997 Identification of a reservoir for HIV-1 in patients on highly active antiretroviral therapy. Science 278:1295–1300. doi:10.1126/science.278.5341.1295. 9360927

[6] Chun TW, Stuyver L, Mizell SB, Ehler LA, Mican JA, Baseler M, Lloyd AL, Nowak MA, Fauci AS 1997 Presence of an inducible HIV-1 latent reservoir during highly active antiretroviral therapy. Proc Natl Acad Sci U S A 94:13193–13197. doi:10.1073/pnas.94.24.13193. 9371822 PMC24285

[7] Lee GQ, Orlova-Fink N, Einkauf K, Chowdhury FZ, Sun X, Harrington S, Kuo HH, Hua S, Chen HR, Ouyang Z, Reddy K, Dong K, Ndung’u T, Walker BD, Rosenberg ES, Yu XG, Lichterfeld M 2017 Clonal expansion of genome-intact HIV-1 in functionally polarized Th1 CD4^+^ T cells. J Clin Invest 127:2689–2696. doi:10.1172/JCI93289. 28628034 PMC5490740

[8] Wagner TA, McLaughlin S, Garg K, Cheung CY, Larsen BB, Styrchak S, Huang HC, Edlefsen PT, Mullins JI, Frenkel LM 2014 HIV latency. Proliferation of cells with HIV integrated into cancer genes contributes to persistent infection. Science 345:570–573. doi:10.1126/science.1256304. 25011556 PMC4230336

[9] Maldarelli F, Wu X, Su L, Simonetti FR, Shao W, Hill S, Spindler J, Ferris AL, Mellors JW, Kearney MF, Coffin JM, Hughes SH 2014 HIV latency. Specific HIV integration sites are linked to clonal expansion and persistence of infected cells. Science 345:179–183. doi:10.1126/science.1254194. 24968937 PMC4262401

[10] Wang Z, Gurule EE, Brennan TP, Gerold JM, Kwon KJ, Hosmane NN, Kumar MR, Beg SA, Capoferri AA, Ray SC, Ho YC, Hill AL, Siliciano JD, Siliciano RF 2018 Expanded cellular clones carrying replication-competent HIV-1 persist, wax, and wane. Proc Natl Acad Sci U S A 115:E2575–E2584. doi:10.1073/pnas.1720665115. 29483265 PMC5856552

[11] Chomont N, El-Far M, Ancuta P, Trautmann L, Procopio FA, Yassine-Diab B, Boucher G, Boulassel M-R, Ghattas G, Brenchley JM, Schacker TW, Hill BJ, Douek DC, Routy J-P, Haddad EK, Sékaly R-P 2009 HIV reservoir size and persistence are driven by T cell survival and homeostatic proliferation. Nat Med 15:893–900. doi:10.1038/nm.1972. 19543283 PMC2859814

[12] Pinzone MR, VanBelzen DJ, Weissman S, Bertuccio MP, Cannon L, Venanzi-Rullo E, Migueles S, Jones RB, Mota T, Joseph SB, Groen K, Pasternak AO, Hwang W-T, Sherman B, Vourekas A, Nunnari G, O’Doherty U 2019 Longitudinal HIV sequencing reveals reservoir expression leading to decay which is obscured by clonal expansion. Nat Commun 10:728. doi:10.1038/s41467-019-08431-7. 30760706 PMC6374386

[13] Sun H, Kim D, Li X, Kiselinova M, Ouyang Z, Vandekerckhove L, Shang H, Rosenberg ES, Yu XG, Lichterfeld M 2015 Th1/17 polarization of CD4 T cells supports HIV-1 persistence during antiretroviral therapy. J Virol 89:11284–11293. doi:10.1128/JVI.01595-15. 26339043 PMC4645661

[14] Wacleche VS, Goulet JP, Gosselin A, Monteiro P, Soudeyns H, Fromentin R, Jenabian MA, Vartanian S, Deeks SG, Chomont N, Routy JP, Ancuta P 2016 New insights into the heterogeneity of Th17 subsets contributing to HIV-1 persistence during antiretroviral therapy. Retrovirology 13:59. doi:10.1186/s12977-016-0293-6. 27553844 PMC4995622

[15] Cosmi L, De Palma R, Santarlasci V, Maggi L, Capone M, Frosali F, Rodolico G, Querci V, Abbate G, Angeli R, Berrino L, Fambrini M, Caproni M, Tonelli F, Lazzeri E, Parronchi P, Liotta F, Maggi E, Romagnani S, Annunziato F 2008 Human interleukin 17-producing cells originate from a CD161^+^CD4^+^ T cell precursor. J Exp Med 205:1903–1916. doi:10.1084/jem.20080397. 18663128 PMC2525581

[16] Takahashi T, Dejbakhsh-Jones S, Strober S 2006 Expression of CD161 (NKR-P1A) defines subsets of human CD4 and CD8 T cells with different functional activities. J Immunol 176:211–216. doi:10.4049/jimmunol.176.1.211. 16365412

[17] Kleinschek MA, Boniface K, Sadekova S, Grein J, Murphy EE, Turner SP, Raskin L, Desai B, Faubion WA, de Waal Malefyt R, Pierce RH, McClanahan T, Kastelein RA 2009 Circulating and gut-resident human Th17 cells express CD161 and promote intestinal inflammation. J Exp Med 206:525–534. doi:10.1084/jem.20081712. 19273624 PMC2699125

[18] Maggi L, Santarlasci V, Capone M, Peired A, Frosali F, Crome SQ, Querci V, Fambrini M, Liotta F, Levings MK, Maggi E, Cosmi L, Romagnani S, Annunziato F 2010 CD161 is a marker of all human IL-17-producing T-cell subsets and is induced by RORC. Eur J Immunol 40:2174–2181. doi:10.1002/eji.200940257. 20486123

[19] Turtle CJ, Swanson HM, Fujii N, Estey EH, Riddell SR 2009 A distinct subset of self-renewing human memory CD8^+^ T cells survives cytotoxic chemotherapy. Immunity 31:834–844. doi:10.1016/j.immuni.2009.09.015. 19879163 PMC2789980

[20] Kryczek I, Zhao E, Liu Y, Wang Y, Vatan L, Szeliga W, Moyer J, Klimczak A, Lange A, Zou W 2011 Human TH17 cells are long-lived effector memory cells. Sci Transl Med 3:104ra100. doi:10.1126/scitranslmed.3002949. PMC3345568 21998407

[21] Muranski P, Borman ZA, Kerkar SP, Klebanoff CA, Ji Y, Sanchez-Perez L, Sukumar M, Reger RN, Yu Z, Kern SJ, Roychoudhuri R, Ferreyra GA, Shen W, Durum SK, Feigenbaum L, Palmer DC, Antony PA, Chan CC, Laurence A, Danner RL, Gattinoni L, Restifo NP 2011 Th17 cells are long lived and retain a stem cell-like molecular signature. Immunity 35:972–985. doi:10.1016/j.immuni.2011.09.019. 22177921 PMC3246082

[22] Fergusson JR, Smith KE, Fleming VM, Rajoriya N, Newell EW, Simmons R, Marchi E, Bjorkander S, Kang YH, Swadling L, Kurioka A, Sahgal N, Lockstone H, Baban D, Freeman GJ, Sverremark-Ekstrom E, Davis MM, Davenport MP, Venturi V, Ussher JE, Willberg CB, Klenerman P 2014 CD161 defines a transcriptional and functional phenotype across distinct human T cell lineages. Cell Rep 9:1075–1088. doi:10.1016/j.celrep.2014.09.045. 25437561 PMC4250839

[23] Alsuliman A, Muftuoglu M, Khoder A, Ahn YO, Basar R, Verneris MR, Muranski P, Barrett AJ, Liu E, Li L, Stringaris K, Armstrong-James D, Shaim H, Kondo K, Imahashi N, Andersson B, Marin D, Champlin RE, Shpall EJ, Rezvani K 2017 A subset of virus-specific CD161(+) T cells selectively express the multidrug transporter MDR1 and are resistant to chemotherapy in AML. Blood 129:740–758. doi:10.1182/blood-2016-05-713347. 27821506 PMC5301823

[24] Huarte E, Cubillos-Ruiz JR, Nesbeth YC, Scarlett UK, Martinez DG, Engle XA, Rigby WF, Pioli PA, Guyre PM, Conejo-Garcia JR 2008 PILAR is a novel modulator of human T-cell expansion. Blood 112:1259–1268. doi:10.1182/blood-2007-12-130773. 18550855 PMC2515140

[25] Deng K, Pertea M, Rongvaux A, Wang L, Durand CM, Ghiaur G, Lai J, McHugh HL, Hao H, Zhang H, Margolick JB, Gurer C, Murphy AJ, Valenzuela DM, Yancopoulos GD, Deeks SG, Strowig T, Kumar P, Siliciano JD, Salzberg SL, Flavell RA, Shan L, Siliciano RF 2015 Broad CTL response is required to clear latent HIV-1 due to dominance of escape mutations. Nature 517:381–385. doi:10.1038/nature14053. 25561180 PMC4406054

[26] Rajasuriar R, Khoury G, Kamarulzaman A, French MA, Cameron PU, Lewin SR 2013 Persistent immune activation in chronic HIV infection: do any interventions work? AIDS 27:1199–1208. doi:10.1097/QAD.0b013e32835ecb8b. 23324661 PMC4285780

[27] Wong JK, Hezareh M, Gunthard HF, Havlir DV, Ignacio CC, Spina CA, Richman DD 1997 Recovery of replication-competent HIV despite prolonged suppression of plasma viremia. Science 278:1291–1295. doi:10.1126/science.278.5341.1291. 9360926

[28] Pallikkuth S, Sharkey M, Babic DZ, Gupta S, Stone GW, Fischl MA, Stevenson M, Pahwa S 2016 Peripheral T follicular helper cells are the major HIV reservoir within central memory CD4 T cells in peripheral blood from chronically HIV-infected individuals on combination antiretroviral therapy. J Virol 90:2718–2728. doi:10.1128/JVI.02883-15. PMC4810658 26676775

[29] Lennartsson J, Ronnstrand L 2012 Stem cell factor receptor/c-Kit: from basic science to clinical implications. Physiol Rev 92:1619–1649. doi:10.1152/physrev.00046.2011. 23073628

[30] Mekori YA, Gilfillan AM, Akin C, Hartmann K, Metcalfe DD 2001 Human mast cell apoptosis is regulated through Bcl-2 and Bcl-XL. J Clin Immunol 21:171–174. doi:10.1023/A:1011083031272. 11403223

[31] Song J, So T, Cheng M, Tang X, Croft M 2005 Sustained survivin expression from OX40 costimulatory signals drives T cell clonal expansion. Immunity 22:621–631. doi:10.1016/j.immuni.2005.03.012. 15894279

[32] Kuo HH, Ahmad R, Lee GQ, Gao C, Chen HR, Ouyang Z, Szucs MJ, Kim D, Tsibris A, Chun TW, Battivelli E, Verdin E, Rosenberg ES, Carr SA, Yu XG, Lichterfeld M 2018 Anti-apoptotic protein BIRC5 maintains survival of HIV-1-infected CD4(+) T cells. Immunity 48:1183–1194.e5. doi:10.1016/j.immuni.2018.04.004. 29802019 PMC6013384

[33] Boyman O, Purton JF, Surh CD, Sprent J 2007 Cytokines and T-cell homeostasis. Curr Opin Immunol 19:320–326. doi:10.1016/j.coi.2007.04.015. 17433869

[34] Surh CD, Sprent J 2008 Homeostasis of naive and memory T cells. Immunity 29:848–862. doi:10.1016/j.immuni.2008.11.002. 19100699

[35] Seddon B, Tomlinson P, Zamoyska R 2003 Interleukin 7 and T cell receptor signals regulate homeostasis of CD4 memory cells. Nat Immunol 4:680–686. doi:10.1038/ni946. 12808452

[36] Bruner KM, Wang Z, Simonetti FR, Bender AM, Kwon KJ, Sengupta S, Fray EJ, Beg SA, Antar AAR, Jenike KM, Bertagnolli LN, Capoferri AA, Kufera JT, Timmons A, Nobles C, Gregg J, Wada N, Ho YC, Zhang H, Margolick JB, Blankson JN, Deeks SG, Bushman FD, Siliciano JD, Laird GM, Siliciano RF 2019 A quantitative approach for measuring the reservoir of latent HIV-1 proviruses. Nature 566:120–125. doi:10.1038/s41586-019-0898-8. 30700913 PMC6447073

[37] Laird GM, Rosenbloom DI, Lai J, Siliciano RF, Siliciano JD 2016 Measuring the frequency of latent HIV-1 in resting CD4(+) T cells using a limiting dilution coculture assay. Methods Mol Biol 1354:239–253. doi:10.1007/978-1-4939-3046-3_16. 26714716

[38] Strain MC, Gunthard HF, Havlir DV, Ignacio CC, Smith DM, Leigh-Brown AJ, Macaranas TR, Lam RY, Daly OA, Fischer M, Opravil M, Levine H, Bacheler L, Spina CA, Richman DD, Wong JK 2003 Heterogeneous clearance rates of long-lived lymphocytes infected with HIV: intrinsic stability predicts lifelong persistence. Proc Natl Acad Sci U S A 100:4819–4824. doi:10.1073/pnas.0736332100. 12684537 PMC153639

[39] Crooks AM, Bateson R, Cope AB, Dahl NP, Griggs MK, Kuruc JD, Gay CL, Eron JJ, Margolis DM, Bosch RJ, Archin NM 2015 Precise quantitation of the latent HIV-1 reservoir: implications for eradication strategies. J Infect Dis 212:1361–1365. doi:10.1093/infdis/jiv218. 25877550 PMC4601910

[40] Hosmane NN, Kwon KJ, Bruner KM, Capoferri AA, Beg S, Rosenbloom DI, Keele BF, Ho YC, Siliciano JD, Siliciano RF 2017 Proliferation of latently infected CD4(+) T cells carrying replication-competent HIV-1: potential role in latent reservoir dynamics. J Exp Med 214:959–972. doi:10.1084/jem.20170193. 28341641 PMC5379987

[41] Bruel T, Schwartz O 2018 Markers of the HIV-1 reservoir: facts and controversies. Curr Opin HIV AIDS 13:383–388. doi:10.1097/COH.0000000000000482. 29846244

[42] Perreau M, Savoye AL, De Crignis E, Corpataux JM, Cubas R, Haddad EK, De Leval L, Graziosi C, Pantaleo G 2013 Follicular helper T cells serve as the major CD4 T cell compartment for HIV-1 infection, replication, and production. J Exp Med 210:143–156. doi:10.1084/jem.20121932. 23254284 PMC3549706

[43] Basdeo SA, Moran B, Cluxton D, Canavan M, McCormick J, Connolly M, Orr C, Mills KH, Veale DJ, Fearon U, Fletcher JM 2015 Polyfunctional, pathogenic CD161^+^ Th17 lineage cells are resistant to regulatory T cell-mediated suppression in the context of autoimmunity. J Immunol 195:528–540. doi:10.4049/jimmunol.1402990. 26062995

[44] Boily-Larouche G, Omollo K, Cheruiyot J, Njoki J, Kimani M, Kimani J, Oyugi J, Lajoie J, Fowke KR 2017 CD161 identifies polyfunctional Th1/Th17 cells in the genital mucosa that are depleted in HIV-infected female sex workers from Nairobi, Kenya. Sci Rep 7:11123. doi:10.1038/s41598-017-11706-y. 28894259 PMC5593931

[45] Fromentin R, Bakeman W, Lawani MB, Khoury G, Hartogensis W, DaFonseca S, Killian M, Epling L, Hoh R, Sinclair E, Hecht FM, Bacchetti P, Deeks SG, Lewin SR, Sekaly RP, Chomont N 2016 CD4^+^ T cells expressing PD-1, TIGIT and LAG-3 contribute to HIV persistence during ART. PLoS Pathog 12:e1005761. doi:10.1371/journal.ppat.1005761. 27415008 PMC4944956

[46] Descours B, Petitjean G, Lopez-Zaragoza JL, Bruel T, Raffel R, Psomas C, Reynes J, Lacabaratz C, Levy Y, Schwartz O, Lelievre JD, Benkirane M 2017 CD32a is a marker of a CD4 T-cell HIV reservoir harbouring replication-competent proviruses. Nature 543:564–567. doi:10.1038/nature21710. 28297712

[47] Hogan LE, Vasquez J, Hobbs KS, Hanhauser E, Aguilar-Rodriguez B, Hussien R, Thanh C, Gibson EA, Carvidi AB, Smith LCB, Khan S, Trapecar M, Sanjabi S, Somsouk M, Stoddart CA, Kuritzkes DR, Deeks SG, Henrich TJ 2018 Increased HIV-1 transcriptional activity and infectious burden in peripheral blood and gut-associated CD4^+^ T cells expressing CD30. PLoS Pathog 14:e1006856. doi:10.1371/journal.ppat.1006856. 29470552 PMC5823470

[48] Shan L, Deng K, Gao H, Xing S, Capoferri AA, Durand CM, Rabi SA, Laird GM, Kim M, Hosmane NN, Yang HC, Zhang H, Margolick JB, Li L, Cai W, Ke R, Flavell RA, Siliciano JD, Siliciano RF 2017 Transcriptional reprogramming during effector-to-memory transition renders CD4(+) T cells permissive for latent HIV-1 infection. Immunity 47:766–775.e3. doi:10.1016/j.immuni.2017.09.014. 29045905 PMC5948104

[49] Simonetti FR, Sobolewski MD, Fyne E, Shao W, Spindler J, Hattori J, Anderson EM, Watters SA, Hill S, Wu X, Wells D, Su L, Luke BT, Halvas EK, Besson G, Penrose KJ, Yang Z, Kwan RW, Van Waes C, Uldrick T, Citrin DE, Kovacs J, Polis MA, Rehm CA, Gorelick R, Piatak M, Keele BF, Kearney MF, Coffin JM, Hughes SH, Mellors JW, Maldarelli F 2016 Clonally expanded CD4^+^ T cells can produce infectious HIV-1 in vivo. Proc Natl Acad Sci U S A 113:1883–1888. doi:10.1073/pnas.1522675113. 26858442 PMC4763755

[50] Bosque A, Famiglietti M, Weyrich AS, Goulston C, Planelles V 2011 Homeostatic proliferation fails to efficiently reactivate HIV-1 latently infected central memory CD4^+^ T cells. PLoS Pathog 7:e1002288. doi:10.1371/journal.ppat.1002288. 21998586 PMC3188522

[51] Gerold JM, Hill AL 2017 Estimating the contribution of proliferation to HIV-infected lymphocyte persistence, abstr 5788. Program Abstr 9th IAS Conf HIV Sci, Paris, France.

[52] Hiener B, Horsburgh BA, Eden JS, Barton K, Schlub TE, Lee E, von Stockenstrom S, Odevall L, Milush JM, Liegler T, Sinclair E, Hoh R, Boritz EA, Douek D, Fromentin R, Chomont N, Deeks SG, Hecht FM, Palmer S 2017 Identification of genetically intact HIV-1 proviruses in specific CD4(+) T cells from effectively treated participants. Cell Rep 21:813–822. doi:10.1016/j.celrep.2017.09.081. 29045846 PMC5960642

