# Supplementary material for: CD161+ CD4+ T Cells Harbor Clonally Expanded Replication-Competent HIV-1 in Antiretroviral Therapy-Suppressed Individuals
Source: mBio. 2019 Oct 8;10(5):e02121-19. doi: 10.1128/mBio.02121-19 (PMC6786872; doi:10.1128/mBio.02121-19)
Supplement: TABLE S1 [file mBio.02121-19-st001.docx]

| **Supplementary Table 1. Fluorochrome-conjugated antibodies used in flow cytometry** | | | | |
| --- | --- | --- | --- | --- |
| **Antigen** | **Specificity** | **Fluorochrome** | **Clone** | **Supplier** |
| **CD3** | Human | FITC | UCHT1 | Bio-legend |
| **CD3** | Human | Percp-cy5.5 | OKT3 | Bio-legend |
| **CD4** | Human | FITC | OKT4/ RPA-T4 | Bio-legend |
| **CD4** | Human | APC | OKT4 | Bio-legend |
| **CD4** | Human | APC-Cy7 | (OKT4) | Bio-legend |
| **CD8** | Human | APC-Fire 750 | RPA-T8 | Bio-legend |
| **CD161** | Human | PE-Cy7 | HP-3G10 | Bio-legend |
| **CD161** | Human | PE | HP-3G10 | Bio-legend |
| **CD27** | Human | APC-Cy7 | O323 | Bio-legend |
| **CCR7** | Human | Brilliant Violet 421 | G043H7 | Bio-legend |
| **CD62L** | Human | APC | DREG-56 | Bio-legend |
| **CD45RA** | Human | PE-Dazzle 594 | HI100 | Bio-legend |
| **CD45RO** | Human | Brilliant Violet 510 | UCHL1 | Bio-legend |
| **HLA-DR** | Human | PE-Dazzle 594 | L243 | Bio-legend |
| **HLA-DR** | Human | FITC | L243 | Bio-legend |
| **CD25** | Human | FITC | BC96 | Bio-legend |
| **CD25** | Human | PE | BC96 | Bio-legend |
| **CD69** | Human | PE | FN50 | Bio-legend |
| **CD69** | Human | FITVC | FN50 | Bio-legend |
| **CD38** | Human | APC-Cy7 | HB-7 | Bio-legend |
| **CCR6** | Human | Brilliant Violet 421 | G034E3 | Bio-legend |
| **CXCR5** | Human | PE | J252D4 | Bio-legend |
| **CCR4** | Human | PE-Cy7 | L291H4 | Bio-legend |
| **CXCR3** | Human | APC | G025H7 | Bio-legend |
| **CXCR4** | Human | PE-Dazzle 594 | 12G5 | Bio-legend |
| **CCR5** | Human | APC | J418F1 | Bio-legend |
| **CCR5** | Human | Percp-Cy5.5 | J418F1 | Bio-legend |
| **CD117** | Human | PE-Cy7 | (104D2) | Bio-legend |
| **TNF-a** | Human | PE-CY7 | MAb11 | Bio-legend |
| **IFN-γ** | Human | APC | 4S.B3 | Bio-legend |
| **IL-17A** | Human | APC | BL168 | Bio-legend |
| **IL-21** | Human | APC | 3A3-N2 | Bio-legend |
| **IL-22** | Human | PE-Cy7 | EH12.2H7 | Bio-legend |
| **HIV-1 P24** | HIV-1 virus | PE | KC57-RD1 | Backman |
| Abbreviations: PE, phycoerythrin; FITC, fluorescein isothiocyanate; APC, allophycocyanin | | | | |
