# Supplementary material for: CD161+ CD4+ T Cells Harbor Clonally Expanded Replication-Competent HIV-1 in Antiretroviral Therapy-Suppressed Individuals
Source: mBio. 2019 Oct 8;10(5):e02121-19. doi: 10.1128/mBio.02121-19 (PMC6786872; doi:10.1128/mBio.02121-19)
Supplement: TABLE S2 [file mBio.02121-19-st002.docx]

| **Supplementary Table 2. Gene-specific primers and probes** | |
| --- | --- |
| **Gene Name** | **Primer Sequence (5'-3')** |
| **Gag** | Forward: ACATCAAGCAGCCATGCAAAT |
|  | Reverse:TCTGGCCTGGTGCAATAGG |
|  | Probe:VIC-CTATCCCATTCTGCAGCTTCCTCATTGATG-TAMRA |
| **Env** | Outer Forward:CAAATTAYAAAAATTCAAAATTTTCGGGTTTATTACAG (HXB2 position 4875–4912) |
|  | Outer Reverse:TGAAGCACTCAAGGCAAGCTTTATTGAGGC (HXB2 position 9607–9636) |
|  | Inner Forward:GATAGACGCGTAGAAAG AGCAGAAGACAGTGGCAATG(HXB2 positions 6191–6227) |
|  | Inner Reverse:CCTTGTGCGGCCGCCTTAAAGGTACCTGAGGTCTGACTGG(HXB2 positions ,.9001–9040) |
| **IL-21** | Forward:TAGAGACAAACTGTGAGTGGTCA |
|  | Reverse:GGGCATGTTAGTCTGTGTTTCTG |
