# Supplementary material for: CD161+ CD4+ T Cells Harbor Clonally Expanded Replication-Competent HIV-1 in Antiretroviral Therapy-Suppressed Individuals
Source: mBio. 2019 Oct 8;10(5):e02121-19. doi: 10.1128/mBio.02121-19 (PMC6786872; doi:10.1128/mBio.02121-19)
Supplement: TABLE S3 [file mBio.02121-19-st003.docx]

**Supplementary Table 3. Gene-specific primers and probes for IPDA**

| **Primer Name** | **Fluorophore,**  **Quencher** | **Sequence (5’-3’)** | **HXB2 coordinates or Gene** |
| --- | --- | --- | --- |
| **ψ** | Forward | CAGGACTCGGCTTGCTGAAG | 692-711 |
| **ψ** | Reverse | GCACCCATCTCTCTCCTTCTAGC | 797-775* |
| **ψProbe** | FAM, MGB | TTTTGGCGTACTCACCAGT | 758-740* |
| **Env** | Forward | AGTGGTGCAGAGAGAAAAAAGAGC | 7736-7759 |
| **Env** | Reverse | GTCTGGCCTGTACCGTCAGC | 7851-7832* |
| **Env_intact** | VIC, MGB | CCTTGGGTTCTTGGGA | 7781-7796 |
| **Env_hypermut** | Unlabeled, MGB | CCTTAGGTTCTTAGGAGC | 7781-7798 |
| **RPP30-2** | Forward | GACACAATGTTTGGTACATGGTTAAAG | RPP30 |
| **RPP30-2** | Reverse | CTTTGCTTTGTATGTTGGCAGAAA | RPP30 |
| **RPP30-2-Probe** | FAM, ZEN/IBFQ | CCATCTCACCAATCATTCTCCTTCCTTC | RPP30 |
| **RPP30-1^a^** | Forward | GATTTGGACCTGCGAGCG | RPP30 |
| **RPP30-1^a^** | Reverse | GCGGCTGTCTCCACAAGT | RPP30 |
| **RPP30-1-Probe^a^** | VIC, ZEN/IBFQ | CTGACCTGAAGGCTCT | RPP30 |

*Reverse compliment

^a^ modified from Massanella et al. Bio-protocol 5(11): e1492
